# Platelet-Rich Plasma Therapy Enhances the Beneficial Effect of Bone Marrow Stem Cell Transplant on Endometrial Regeneration

**DOI:** 10.3389/fcell.2020.00052

**Published:** 2020-02-21

**Authors:** Ying Zhou, Huaxiang Shen, Yuelin Wu, Xiaobo Zhao, Jindan Pei, Zhengqian Mou, Jinhua Dong, Xiaolin Hua

**Affiliations:** ^1^Department of Obstetrics, Shanghai First Maternity and Infant Hospital, Tongji University School of Medicine, Shanghai, China; ^2^Department of Obstetrics, Jiaxing Maternity and Child Health Care Hospital, Jiaxing, China; ^3^Department of Obstetrics and Gynecology, Xinhua Hospital, Shanghai Jiao Tong University School of Medicine, Shanghai, China

**Keywords:** bone marrow stem cells, platelet-rich plasma, uterine endometrium, insulin-like growth factor-1, uterine horn damage

## Abstract

This study aimed to investigate the potential effect of platelet-rich plasma (PRP) therapy on treatment using bone marrow stem cell (BMSC) transplant for uterine horn damage, and to reveal the potential underlying molecular mechanism. Uterine horn damage was established in a rat model, which can be repaired by transplant using BMSCs receiving control or PRP treatment. Immunohistochemistry was conducted to evaluate thickness and expression of α-SMA and vWF in the regenerated endometrium tissues. mRNA and proteins of insulin-like growth factor-1 (IGF-1) and interleukin-10 (IL-10) were measured both in the regenerated endometrium tissues and in cultured BMSCs to evaluate the effect of PRP treatment on their expression. Enzyme-linked immunosorbent assay was employed to measure the secretory levels of IL-10 in cultured BMSCs. Multi-differentiation assays were performed to address the effect of PRP treatment on tri-lineage potential of cultured BMSCs. Chromatin immunoprecipitation and luciferase reporter assays were applied to analyze NF-κB subunit p50 binding on IL-10 promoter and the resulted regulatory effect. PRP treatment significantly improved the efficacy of BMSC transplant in repairing uterine horn damage of rats, and elevated IGF-1 and IL-10 expression in regenerated endometrium tissues and cultured BMSCs, as well as enhanced tri-lineage differentiation potential of BMSCs. On the other hand, p50 inhibition and silencing suppressed the PRP-induced expression and secretion of IL-10 without affecting IGF-1 in the BMSCs. Furthermore, p50 was able to directly bind to IL-10 promoter to promote its expression. Data in the current study propose a working model, where PRP therapy improves endometrial regeneration of uterine horn damage in rats after BMSC transplant therapy, likely mediated through the NF-κB signaling pathway subunit p50 to directly induce the expression and production of IL-10.

## Introduction

The uterine endometrium, a term referring to the inner epithelial layer in the uterus and its mucous membrane, contains a basal layer and a functional layer. The basal layer is where regeneration occurs exclusively, while the functional layer presents a cyclic slough and renewal over the menstrual period (Maruyama and Yoshimura, 2008). Damages associated with endometrium’s basal layer have an obvious impact on the regenerative disorder of epithelium, scar formation, fibrosis and intrauterine adhesion, leading to infertility and amenorrhea (Yu et al., 2008). Primary clinical treatment includes hysteroscope adhesiolysis by adjuvant estrogen therapy, together with placing stents with a expandable balloon or intrauterine equipment after surgery, which shows certain therapeutic effect on mild and moderate cases (March, 2011). Nevertheless, severe cases present unsatisfactory prognosis and the recurrent adhesion incidence rate ranges from 20–62.5%, which is usually related to irreversible impairment in terms of fertility and receptivity (Deans and Abbott, 2010). In this context, enhancing proliferation of endometrial cells and subsequent reconstruction of the endometrial structure, as well as restoring functions of the endometrium, are the key solution to endometrial damage repair in order to improve pregnancy rate.

Bone marrow-derived mesenchymal stem cells (BMSCs) exhibit multi-differential potency of being derived into various types of mature cells, which have therefore been applied in regenerative medicine to treat complex tissue damages (Tatullo et al., 2015). In a recent research, minimally invasive BMSC transplant can be used to effectively treat uterine prolapse in a rat model (Jin et al., 2016). In another similar study, BMSCs can facilitate endometrial stromal cell proliferation and endometrium accretion after differentiation into endometrial epithelial cells, thereby improving pregnancy rate in an animal endometrial damage model (Alawadhi et al., 2014). After transplant, BMSCs utilize the autocrine and paracrine pathways to potentially activate multiple cell types to reconstruct the damaged uterine endometrium (Ding et al., 2014). In addition, a recent report has raised the possibility that endometrial stem cells likely originate from BMSCs (Alcayaga-Miranda et al., 2015). This speculation is in agreement with the theory that, BMSCs exhibit dual functions in the repair of endometrium: (1) directly differentiating into endometrial epithelial cells, and (2) activating resident endometrial stem cells. At the same time, only a small proportion of bone marrow cells are stem cells, and their differentiation capacity declines with age (Alcayaga-Miranda et al., 2015), therefore direct injection of BMSCs presents an obviously lower therapeutic effect compared with the current control, which raises in two major clinical challenges: (1) improving the generation, attachment, proliferation and differentiation of BMSCs, and (2) promoting the proliferation of differentiated epithelial cells to achieve ultimate recovery of endometrium.

Platelet-rich plasma (PRP) refers to a method to concentrate platelet in plasma to a higher level than in the whole blood (Malanga and Goldin, 2014). PRP contains multiple beneficial factors to augment healing, therefore clinical applications of PRP therapy have soared in the last 10 years, especially in spine, sport, and musculoskeletal medicine (Wu et al., 2016). In particular, PRP contains cell adhesion molecules and chemotactic properties that help recruit BMSCs to the repair site (Boswell et al., 2012). For instance, in a meniscal repair rabbit model *in vivo*, a PRP scaffold augmented BMSCs for a complete tissue repair (Zellner et al., 2010).

Recently, our group has established that transplant of insulin-like growth factor-1 (IGF-1) overexpressing BMSCs improved functional regeneration of uterine endometrium in a rat model of uterine horn damage (Wang et al., 2018). Interestingly, PRP has been shown to induce various growth factors involved in the healing process, including IGF-1 (Boswell et al., 2012). These findings together have prompted us to assess whether PRP might be potentially able to enhance the beneficial effect of BMSC transplant on endometrial regeneration of uterine horn damage.

## Materials and Methods

### Rat BMSC Isolation and Culture

We isolated BMSCs of rat and then cultured them based on steps described previously (Ding et al., 2014). Briefly, female rats at the age of 2 months were sacrificed by overdosing with ketamine. We firstly isolated the tibias and femora and used phosphate-buffered saline (PBS, Gibco, Grand Island, NY, United States) to completely wash them, followed by carefully removing the extraossial tissues and muscles. Dulbecco’s modified Eagle’s medium (DMEM, Gibco, United States) with low glucose was used to completely wash marrow cells in bone cavities. The resulting lavage was firstly passed through a cell strainer (100 μm, BD Bioscience, Franklin Lakes, NJ, United States), and was then centrifuged. Single cell suspension was collected in LG-DMEM supplemented with 1 × insulin-transferrin-selenium (Gibco, United States), 12.5% fetal bovine serum (FBS, Gibco, United States), 1% penicillin-streptomycin-glutamine (Gibco, United States) and 10 ng/mL basic fibroblast growth factor (Gibco, United States). We seeded these isolated cells into 100 mm diameter culture dishes and kept them in a humid incubator with 5% CO_2_ at 37°C. We then replaced fresh medium every other day and used log-phase cells of passage 3–5 for experiments later.

### PRP Preparation

Platelet-rich plasma preparation followed previously established procedures (Liao, 2019). Briefly, 2% isoflurane was used to perform general anesthesia on Aprague-Dawley rats. We took 8 mL of blood from each rat by decapitation and transferred them into a centrifuge tube which contained 2 ml of acid citrate dextrose solution for preventing blood clotting. Every centrifuge tube containing 10 ml of whole blood received 10 min centrifugation at 4000 rpm to separate plasma. We separated the precipitated platelets at the bottom of centrifuge tubes, and the supernatant was collected as PRP. The platelet counts in the PPP were calculated by a hematology analyzer, which was freshly diluted at 5000 count/mL in culture media for BMSC treatment.

### Multi-Differentiation Assays

The differentiation potential of BMSCs in terms of adipogenesis, chondrogenesis and osteogenesis, with or without PRP treatment, was assessed by multi-differentiation assay. BMSCs were maintained in the following conditioned media for 14 days: (1) adipogenic induction medium (DMEM, 100 mM indomethacin, 20% FBS, and 0.5 mM isobutylmethylxanthine); (2) chondrogenic induction medium (DMEM, 0.2 mM ascorbic 2-phosphate, 20% FBS, and 10 mM glycerol 2-phosphate); (3) osteogenic induction medium (DMEM-low glucose, 50 mg/ml ascorbic 2-phosphate, 20% FBS, 50 mg/ml insulin-transferrin-selenious acid mix, and 100 mg/ml sodium pyruvate). After 14 days, adipogenic differentiation was assessed based on Oil Red O (Millipore, Billerica, MA, United States) staining, chondrogenesis was evaluated using Alcian Blue (Millipore, Billerica, MA, United States) staining, and osteogenesis was measured with the use of Alizarin Red solution (Millipore, Billerica, MA, United States).

### Rat Uterine Horn Damage Model, BMSC Transplant and PRP Therapy

Experimenters collected vaginal smears in early morning on a daily basis, and evenly divided a total of 32 female Aprague-Dawley rats, with average body weight of 250 g ∼ 300 g and on four consecutive estrus cycles, in a random manner into 4 groups: sham operated group (sham), spontaneous repair group (model), Model + BMSCs group, Model + BMSCs + PRP group. For the establishment of the uterine horn damage model, rats firstly received anesthesia treatment using 5% isoflurane, and maintained at 1% during treatment. The anesthetized rat was shaved, and skin was sterilized using 70% ethanol solution. The renal portion was incised, and the fat pad near ovary was drawn to expose and clip the uterus horn, and a low abdominal midline incision was performed on the uterine horn. Experimenters resected a segment in the size of 1.5 cm × 0.5 cm from the uterine horn and maintained side of the mesometrium intact. The opened skin and muscle layer were sutured using absorbable suture, and the skin was sterilized with 10% povidone-iodine. After surgery, rats were covered by warm blankets and remained under careful supervision to monitor health and body conditions. Specific to sham operation (Sham group), the horns of uterine were maintained complete following an abdominal midline incision. In spontaneous repair group (Model group), gelatin scaffolds without BMSCs was used for replacing excised segments, and no further treatment was performed on uterine horns, which had obvious defect with complete hemostasis. Specific to the Model + BMSCs group, we sutured gelatin scaffolds with 5 × 10^5^ BMSCs for replacing excised segments. Specific to the Model + BMSCs + PRP group, we used the PRP (5000 count/mL) to immerse gelatin scaffolds with the BMSCs and then sutured them for excised segments replacement. Specific to the Model + BMSCs/si-p50 + PRP group, we used the PRP (5000 count/mL) to immerse gelatin scaffolds with stable p50-silenced BMSCs and then sutured them for excised segments replacement. All the rats received intramuscular injection twice a day with penicillin for three continuous days after the surgery for systematic infection prevention. Gelatin scaffold was employed in the current study as it exhibits good biocompatibility and biodegradability in stem cell transplant according to various previous studies (Fu et al., 2014; Zeng et al., 2015; Bhowmick et al., 2016).

### Histological Examination

Experimenters resected uterine horn’s regenerative region and used 4% paraformaldehyde to fix them overnight, followed by using graded alcohol for dehydration as well as embedding in paraffin. The microtome (Leica RM2255, German) was used to slice tissues into 5 μm sections in a transverse manner. The anti-von Willebrand factor antibody (vWF, Abcam, ab6994, 1: 10000) and anti-α-smooth muscle actin antibody (α-SMA, Abcam, ab5694, 1:200) were used to perform immunostaining on parts of tissues, respectively. The Image-Pro Plus software was used to estimate the actin density of muscle based on the percentage taken by α-SMA-positive area, and light microscope was used to count the capillary vessels in at least three random fields.

### *In vitro* Treatment of BMSCs With PRP and p105sr

*In vitro* treatment of the BMSCs with PRP followed previously established procedures (Kim et al., 2017). Briefly, experimenters seeded BMSCs with 1 × 10^6^ cells in a T75 flask and kept them in an incubator containing 5% CO_2_ at 37°C. 500 μl PRP was added into the culture after 24 h incubation with DMED containing 12.5% FBS. For the negative control group, experimenters only seeded the BMSCs with the same density in a T75 flask in the presence of DMEM containing 12.5% FBS. Culture flasks underwent a 7-day incubation at 37°C in a wet atmosphere that contained 5% CO_2_. In the abovementioned conditions, we changed fresh medium every 2 days. p105sr is a plasmid expressing NF-κB subunit containing a deletion in the cleavage domain, preventing signal-induced degradation (Fu et al., 2004).

### Reverse Transcription Polymerase Chain Reaction (RT-PCR)

Trizol reagent (Invitrogen, Carlsbad, CA, United States) was used to extract total RNA from the indicated cells or tissues based on the instructions of manufacturer. BioAnalyzer 2100 (Agilent, Santa Clara, CA, United States) used to determine the RNA quality and quantity. The High-Capacity cDNA Reverse Transcription Kit (ThermoFisher, Waltham, MA, United States) was used for the synthesis of cDNA. RT-PCR was carried out using the GoTaq Green Master Kit (Promega, Madison, WI, United States) following recommendations of manufacturer. Primers used in the current study were listed in Table 1. The 2^–ΔΔ*Ct*^ method was adopted to calculate relative expression of the target gene, which was then normalized to *GAPDH*.

**TABLE 1:** Primers used in the current study.

*IGF-1* Forward	5′-AAA TCA GCA GCC TTC CAA CTC-3′
*IGF-1* Reverse	5′-GCA CTT CCT CTA CTT GTG TTC TT-3′
*IL-10* Forward	5′-AGC CTT ATC GGA AAT GAT CCA GT-3′
*IL-10* Reverse	5′-GGC CTT GTA GAC ACC TTG GT-3′
*p50* Forward	5′-GGA GGC ATG TTC GGT AGT GG-3′
*p50* Reverse	5′-CCC TGC GTT GGA TTT CGT G-3′
*GAPDH* Forward	5′-GGA GCG AGA TCC CTC CAA AAT-3′
*GAPDH* Reverse	5′-GGC TGT TGT CAT ACT TCT CAT GG-3′

### Western Blot

Preparation of cell lysates was performed on ice in radioimmunoprecipitation assay lysis buffer. Cell lysate supernatant was collected after centrifugation, followed quantification using BCA Protein Assay Kit (ThermoFisher, United States). Sodium dodecyl sulfate polyacrylamide gel electrophoresis was adopted to resolve protein samples, followed by transferring to polyvinylidene difluoride (PVDF) membrane on the ice. 5% skim milk was used to block the PVDF membrane in TBST buffer for 1 h at room temperature with indicated primary antibodies (anti-p50, ab32360, Abcam, 1:1000; anti-interleukin-10 (IL-10), ab34843, Abcam, 1:1000; anti-IGF-1, ab9572, Abcam, 1:1000; anti-GAPDH, ab9485, Abcam, 1:1000) at 4°C overnight. After using TBST to wash the membrane for five times, PVDF membrane was incubated for 1 h at room temperature with appropriate horseradish peroxidase-conjugated secondary antibodies (anti-mouse, ab97046, Abcam, 1:5000; anti-rabbit, ab6721, Abcam, 1:5000). TBST was used to thoroughly wash the membrane for five times. Enhanced chemiluminescence kit (ECL, Millipore, Billerica, MA, United States) was used to visualize the protein bands, and ChemiDoc Touch Imaging System (Bio-Rad, Hercules, CA, United States) was used to obtain images.

### Enzyme-Linked Immunosorbent Assay (ELISA)

The commercial Rat IGF-1 Quantikine ELISA Kit (MG100, R&D SYSTEMS, United States) was applied for the detection of secretory IGF-1 following instructions of manufacturer. In brief, we collected conditioned medium from the indicated cell culture, which was centrifuged to remove cell debris. 50 μL of assay diluent was mixed with 50 μL of supernatant in each 96-well plate, which then underwent 2 h of incubation at room temperature on an aclinic orbital microplate shaker. After complete aspiration of the supernatant, wells were thoroughly washed for five times. After adding substrate solution into each well, the reaction underwent 30 min incubation in dark, and 100 μL stop solution was used to terminate the reaction. TECAN Infinite 200 PRO (TECAN, United States) was used to record absorbance at 450 nm with OD 570 nm as the reference.

### Stable p50 shRNA Knockdown

Sigma-Aldrich (St. Louis, MO, United States) provided the MISSION non-Target shRNA Control (SHC016) and the MISSION Lentiviral shRNA kit against p50 (SHCLNG-NM_008689). Both lentiviral plasmids were packaged into the lentivirus in 293T cells based on instructions of manufacturer. We seeded log-phase BMSCs into 6-well plates and used harvested p50 shRNA lentivirus particles to infect them for 24 h. Infected BMSCs were then transferred into 100 mm petri dish, followed by puromycin selection to maintain stable p50 knockdown.

### Chromatin Immunoprecipitation (ChIP) Assay

ChIP assay was performed using a commercial kit from Abcam (ChIP Kit ab500). Briefly, cells were cross-linked in 37% formaldehyde for 20 min at room temperature with gentle rotation. Glycine was added to a final concentration of 0.125 mM, followed by rinsing in ice-cold PBS. Cells were then resuspended in lysis buffer and sonication to shear DNA, followed by centrifugation at 8,000 *g* for 10 min at 4°C to remove cell debris. The supernatant was then immunoprecipitated using protein A beads attached with anti-p50 antibody (ab32360, Abcam) or IgG control at 4°C overnight with gentle rotation. Immunoprecipitated beads were then washed extensively, followed by reverse crosslinking with 200 mM NaCl. DNA was purified using a PCR purification kit, which was then subject to qPCR analysis (with 1/10 input). All sample data were normalized to IgG control.

### Luciferase Reporter Assay

The wild-type or mutated 5′-promoter sequence of IL-10 was inserted into pGL4 dual luciferase reporter vectors, as Wt-Lcf and Mut-Lcf, respectively, by Vivabiotech (Shanghai, China). Luciferase reporter plasmid was co-transfected with control vector or p50 into BMSCs. Relative luciferase activity was measured 24 h after transfection by Luciferase Assay System (Promega, Madison, WI, United States). Ectopic expression of p50 was achieved using the pCMV4/p50 plasmid (#21965; Addgene, Watertown, MA, United States).

### Statistical Analysis

All data used in the study were obtained from at least three independent biological repeats in triplicates. SPSS 23.0 was used to analyze the results, which were presented as mean ± standard deviation (SD). Multiple comparison was conducted by one-way analysis of variance (ANOVA), followed by the Turkey *post hoc* test. Statistical significance was calculated as *P*-values, with *P* < 0.05 indicating statistical difference.

## Results

### BMSC Isolation and Rat Uterine Horn Damage Model

**FIGURE 1:**
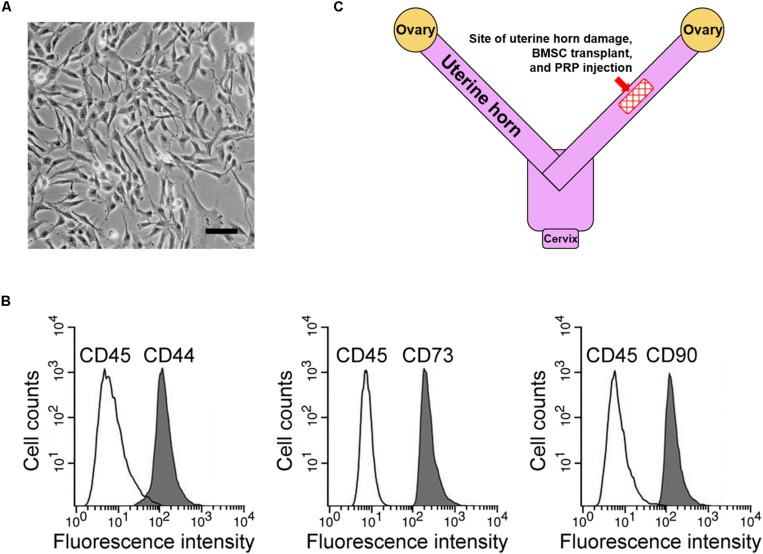
BMSC isolation and rat uterine horn damage model. **(A)** Representative image of cultured BMSCs, scale bar 100 μm. **(B)** Flow cytometry identified BMSC positive surface markers CD44, CD73, and CD90, and negative surface marker CD45. **(C)** Illustration of the rat model, showing the site for uterine horn damage, BMSC transplant, and PRP therapy.

First, rat BMSCs were isolated as described in the methods (Figure 1A). Based on our flow cytometry analysis data, these cells tested positive for three surface markers, namely CD44, CD73, and CD90, while negative for the surface marker CD45 (Figure 1B), consistent with a previous report on BMSC surface marker signatures (Boxall and Jones, 2012). Next, potential therapeutic effects of BMSC transplant to treat the rat uterine horn damage model (Figure 1C) was investigated.

### PRP Therapy Enhances Effect of BMSC Transplant in Treating Injured Rat Uterus

**FIGURE 2:**
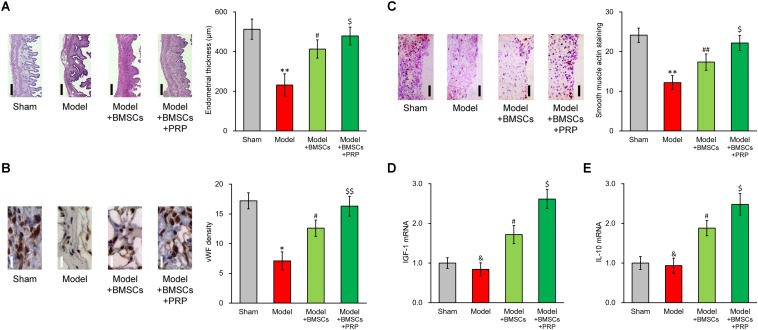
PRP therapy enhances BMSC transplant in treating injured rat uterus. Rats were divided into sham, model, model receiving only BMSC transplant (Model + BMSCs) and model receiving both BMSC transplant and PRP therapy (Model + BMSCs + PRP), with eight rats per group. 28 days after the treatment, **(A)** representative histological images (left side, scale bar 100 μm) and bar graph showing endometrial thickness in the regenerative uterine horns, **(B)** representative histological images (left side, scale bar 20 μm) and bar graph showing neovascularization measured as vWF staining density in the newly regenerated uterine endometrial, **(C)** representative histological images (left side, scale bar 50 μm) and bar graph showing smooth muscle regeneration measured as smooth muscle actin staining, were assayed in the experimental animals. **(D,E)** mRNA levels of IGF-1 **(D)** and IL-10 **(E)** were examined in regenerated endometrium samples 28 days after the treatment. Values are mean ±SD (*n* = 8 each group). ^∗∗^*P* < 0.01, ^∗^*P* < 0.05, &*P* > 0.05, vs. sham. #*P* < 0.05, ##*P* < 0.01, vs. both sham and model. $*P* < 0.05, $$*P* < 0.01, vs. both model and model + BMSCs.

We examined endometrium regeneration in the injured rat uterus 28 days after surgery. Figure 2A demonstrates representative histological images showing endometrial sections in the model group, which exhibited a remarkably lower endometrial thickness than rats in the sham group, indicating that the pathologic process of endometrium impairment was well established in our model. The endometrial thickness was greatly increased after BMSC transplant into the rat uterus, further suggesting that the BMSC transplant therapy was effective for restoring the uterine horn damage. Importantly, PRP therapy obviously further improved the healing process compared with BMSC transplant alone, evidenced by the nearly completely restored endometrial thickness.

Furthermore, immunostaining was performed on specific markers to assess the regeneration of smooth muscles and neovascularization during recovery process. vWF staining indicated an obvious angiogenesis process following BMSC transplant, and was further enhanced by PRP therapy (Figure 2B). Regeneration of smooth muscle also showed an obvious improvement following BMSC transplant, and was further enhanced by PRP therapy as well (Figure 2C). These *in vivo* data demonstrated that PRP therapy was beneficial in promoting the recovery of injured uterus using BMSC transplant.

### PRP Treatment Induces IGF-1 and IL-10 Production of BMSCs *in vitro*

**FIGURE 3:**
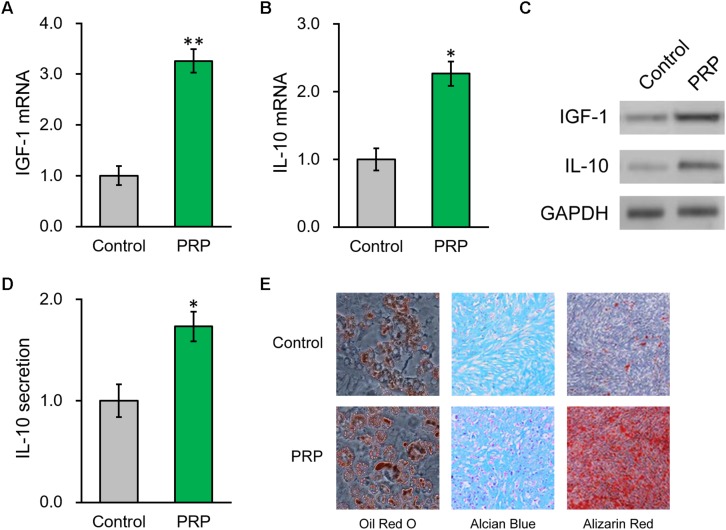
PRP treatment induces IGF-1 and IL-10 production from of BMSCs *in vitro.* **(A)** IGF-1 mRNA, **(B)** IL-10 mRNA and **(C)** their protein levels in control BMSCs and BMSCs treated with PRP. **(D)** IL-10 levels in media culturing only BMSCs and BMSCs + PRP treatment. **(E)** At day 14 after differentiation induction with or without PRP treatment, the extents of adipogenesis, chondrogenesis and osteogenesis were evaluated by Oil Red O, Alcian Blue, and Alizarin Red S staining assays, respectively. Values are mean ±SD at least three independent biological repeats in triplicates. ^∗^*P* < 0.05, ^∗∗^*P* < 0.01, vs. control.

Upon observing the promotional effect of PRP therapy in the rat uterus damage model, we naturally wanted to investigate the effect of PRP therapy on expression status of IGF-1 in the BMSCs. As expected, in the regenerated endometrium samples, the group receiving BMSC transplant showed elevated IGF-1 and IL-10 mRNA levels (Figures 2D, 3E, Model + BMSCs), which were further increased in the group transplanted with PRP-treated BMSCs (Figures 2D,E, Model + BMSCs + PRP).

We then took advantage of a PRP-stem cell system reported previously (Kim et al., 2017) and found that, in the BMSCs treated with PRP, endogenous mRNA levels of IGF-1 and IL-10 were also greatly increased compared to BMSCs cultured alone (Figures 3A,B). Consistently, their protein levels in BMSCs were also significantly elevated by PRP treatment (Figure 3C and Supplementary Figure S1). Considering the role of IL-10 as a secretory cytokine, its levels in the culture media of the BMSCs, in absence or presence of PRP, were further assessed. According to the ELISA results, IL-10 secretion was significantly stimulated by PRP treatment (Figure 3D). Furthermore, adipogenesis, chondrogenesis and osteogenesis differentiation assays were performed in BMSCs with or without PRP treatment, and the results indicated that PRP treatment increased the tri-lineage potential of BMSCs (Figure 3E). On the other hand, PRP treatment didn’t affect viability or proliferation of BMSCs (data not shown). On that account, our results demonstrated that the role of PRP to stimulate IGF-1 expression, IL-10 expression and secretion, as well as tri-lineage differentiation potential of BMSCs, played major roles responsible for its enhancement on BMSC transplant in treating injured rat uterus.

### NF-κB p50 Subunit Inhibition Abolishes the PRP-Induced IL-10 Expression and Secretion in BMSCs

**FIGURE 4:**
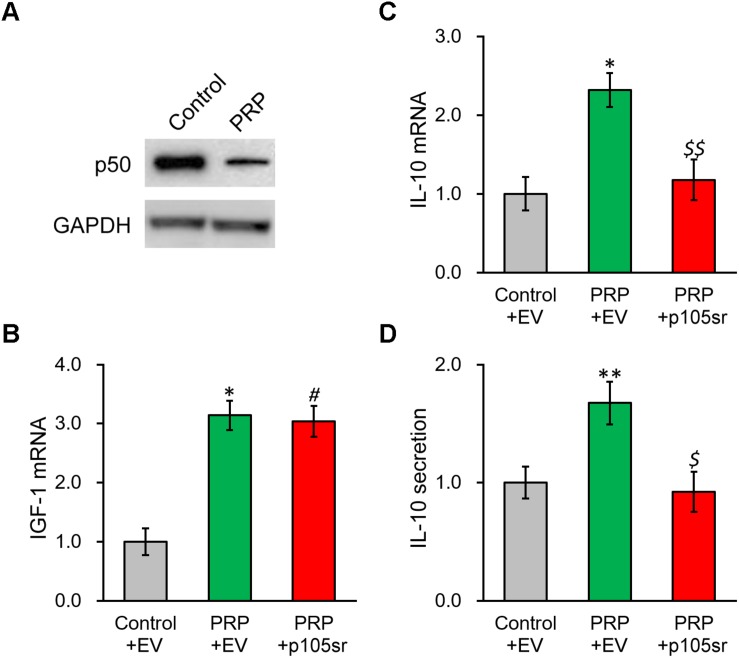
Inhibition of NF-κB p50 subunit abolishes PRP-induced expression and secretion of IL-10 in BMSCs. **(A)** p50 protein level was examined in control BMSCs and BMSCs treated with PRP. **(B)** IGF-1 mRNA, **(C)** IL-10 mRNA and **(D)** IL-10 secretion in media were examined in control BMSCs transfected with empty vector (control + EV), BMSCs treated with PRP and transfected with empty vector (PRP + EV), and BMSCs treated with PRP and transfected with p50 inhibitor p105sr (PRP + p105sr). Values are mean ±SD at least three independent biological repeats in triplicates. ^∗∗^*P* < 0.01, ^∗^*P* < 0.05, vs. control + EV. #*P* < 0.05 vs. control + EV and not significant vs. PRP + EV. $, $$ not significant vs. control + EV and *P* < 0.05, 0.01, respectively, vs. PRP + EV.

We next interrogated the molecular mechanism underlying PRP-induced IL-10 expression and secretion in BMSCs. As NF-κB signaling was reported to affect the regulation of IL-10, we checked the NF-κB subunit p50 in BMSCs following PRP treatment, as well as its potential role in modulating IL-10 in this process. Figure 4A and Supplementary Figure S2 show that p50 protein level exhibited an obvious reduction in PRP-treated BMSCs compared with the control group, suggesting that the NF-κB pathway was activated via the subunit p50, hence was likely involved in the regulation of IL-10. To this end, the p50-specific inhibitor, p105sr, was transfected into the BMSCs treated with PRP. According to the RT-PCR results in Figure 4B, p105sr inhibitor treatment did not affect the expression of IGF-1, placing the function of p50 downstream of IGF-1. On the other hand, p105sr inhibitor treatment greatly lowered the secretion and expression of IL-10 (Figures 4C,D), which highlighted that NF-κB subunit p50 could greatly stimulate IL-10 production following PRP treatment.

### NF-κB p50 Subunit Knockdown Abolishes the PRP-Induced IL-10 Expression and Secretion in BMSCs

**FIGURE 5:**
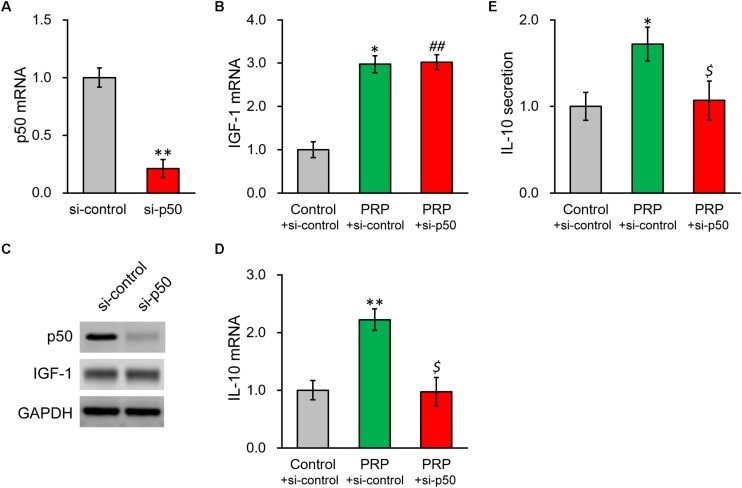
Knockdown of NF-κB p50 subunit abolishes PRP-induced expression and secretion of IL-10 in BMSCs. **(A)** p50 mRNA, **(B)** IGF-1 mRNA, and **(C)** their protein levels were examined in control knockdown BMSCs (si-control) and p50 knockdown BMSCs (si-p50). **(D)** IL-10 mRNA levels were examined in control knockdown BMSCs (si-control), control knockdown BMSCs treated with PRP (PRP + si-control), and p50 knockdown BMSCs treated with PRP (PRP + si-p50). **(E)** IL-10 levels in media culturing control knockdown BMSCs (si-control), control knockdown BMSCs treated with PRP (PRP + si-control), and p50 knockdown BMSCs treated with PRP (PRP + si-p50). Values are mean ± SD at least three independent biological repeats in triplicates. ^∗∗^*P* < 0.01, ^∗^*P* < 0.05, vs. si-control. ##*P* < 0.05 vs. si-control and not significant vs. PRP + si-control. $ not significant vs. si-control and *P* < 0.05 vs. PRP + si-control.

To further confirm our above conclusion, NF-κB p50 subunit was silenced via a specific shRNA. The knockdown efficiency was verified by both RT-PCR and Western blot (Figures 5A,C), where p50 mRNA was reduced to ∼20% of control. As expected, p50 knockdown failed to impact IGF-1 at transcript or protein level (Figures 5B,C and Supplementary Figure S3). In contrast, p50 knockdown nearly completely repressed the PRP-induced IL-10 expression and secretion in BMSCs (Figures 5D,E), confirming the role of NF-κB subunit p50 was indeed at the downstream of IGF-1 in the PRP-induced upregulation of IL-10.

### NF-κB p50 Subunit Induces IL-10 Expression by Directly Binding to Its Promoter Region

**FIGURE 6:**
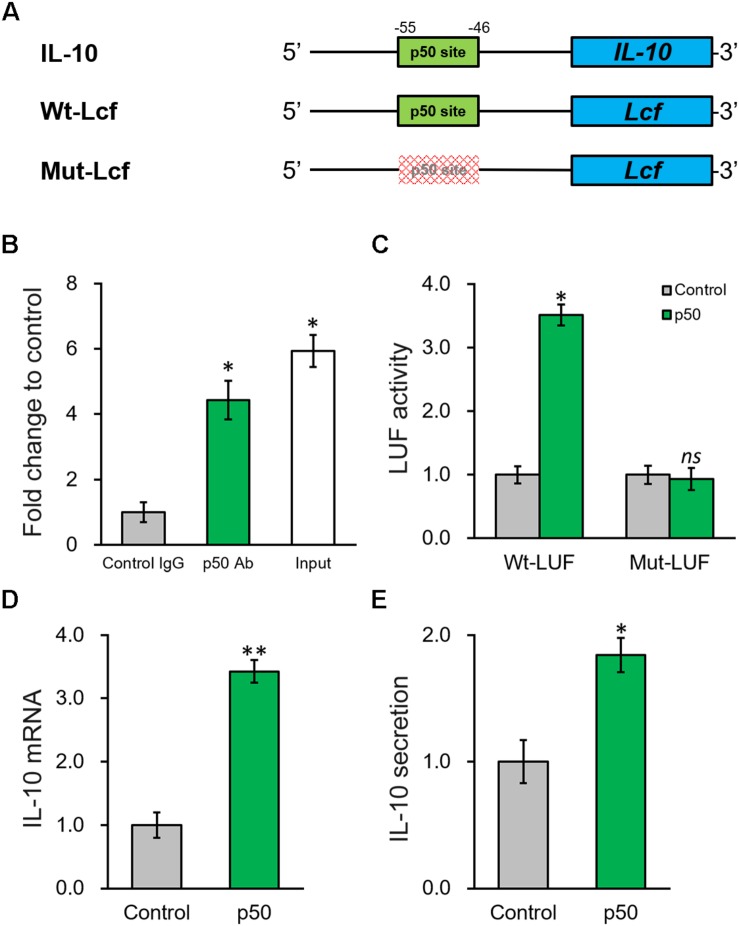
NF-κB p50 subunit induces IL-10 expression by directly binding to its promoter region. **(A)** The putative p50 binding site was identified in the promoter region of IL-10. Wild type (Wt-LUF) and mutated (Mut-LUF) sequences from IL-10 promoter were cloned, respectively, to the 5′-prime of luciferase reporter gene (LUF). **(B)** Binding of p50 to the putative binding site in IL-10 promoter were analyzed by ChIP assay using control IgG and p50 antibody (Ab), respectively. **(C)** Luciferase activities of Wt-LUF or Mut-LUF constructs described in **(A)** were measured in BMSCs transfected with control vector or p50, respectively. **(D,E)** Levels of IL-10 mRNA with representative gel images of PCR products shown below the bar graph **(D)**, and in media **(E)** were examined in BMSCs transfected with control vector or p50, respectively. Values are mean ±SD at least three independent biological repeats in triplicates. ^∗∗^*P* < 0.01, ^∗^*P* < 0.05, ns, not significant, vs. respective control.

According to the results above, knowing p50 could essentially induce the secretion and expression of IL-10, it is still necessary to further define the potential molecular regulatory mechanism responsible. By inspecting the promoter sequence of IL-10, we found a proximal putative binding site of p50 (Figure 6A). ChIP assay was employed to evaluate direct p50 binding to this predicted sequence on IL-10 promoter. Indeed, the putative p50 binding sequence on IL-10 promoter in the p50-immunoprecipitate exhibited approximately 5-fold enrichment compared with the IgG control (Figure 6B). Next, p50 direct regulation on IL-10 was investigated by constructing a wild type or mutated IL-10 promoter sequence at the upstream of a luciferase reporter open reading frame (Figure 6A). An obvious simulating effect of ectopic p50 expression was observed on the luciferase activity compared with control, while the inducing effect was eliminated by the mutated p50-binding sequence (Figure 6C). Similarly, induced by the ectopic expression of p50 in BMSCs, endogenous IL-10 expression and secretion were significantly up-regulated as well (Figures 6D,E). Based on our data, p50 could activate IL-10 transcription in BMSCs following PRP treatment through directly binding to its promoter, and subsequently facilitating the function and secretion of the anti-inflammatory cytokine IL-10.

### p50 Is Needed to Enhance Effect of PRP on BMSC Transplant in the Treatment of Injured Rat Uterus

**FIGURE 7:**
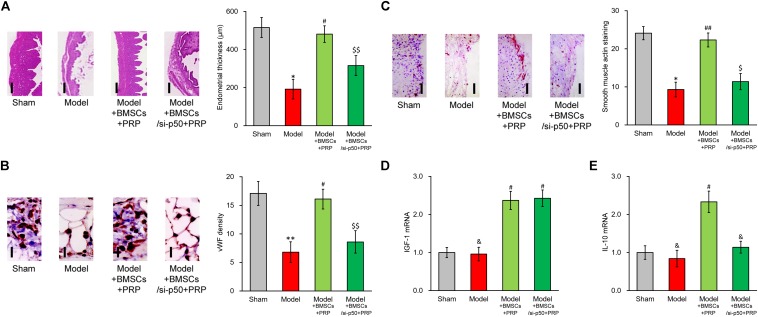
Enhancing effect of PRP therapy on BMSCs transplant in treating injured rat uterus requires p50. Rats were divided into sham, model, model receiving control BMSC transplant and PRP therapy (Model + BMSCs + PRP) and model receiving p50-silenced BMSC transplant and PRP therapy (Model + BMSCs/si-p50 + PRP), with 8 rats per group. 28 days after the treatment, **(A)** representative histological images (left side, scale bar 100 μm) and bar graph showing endometrial thickness in the regenerative uterine horns, **(B)** representative histological images (left side, scale bar 20 μm) and bar graph showing neovascularization measured as vWF staining density in the newly regenerated uterine endometrial, **(C)** representative histological images (left side, scale bar 50 μm) and bar graph showing smooth muscle regeneration measured as smooth muscle actin staining, were assayed in the experimental animals. **(D,E)** mRNA levels of IGF-1 **(D)** and IL-10 **(E)** were examined in regenerated endometrium samples 28 days after the treatment. Values are mean ±SD (*n* = 8 each group). ^∗∗^*P* < 0.01, ^∗^*P* < 0.05, &*P* > 0.05, vs. sham. #*P* < 0.05, ##*P* < 0.01, vs. both sham and model. $*P* < 0.05, $$*P* < 0.01, vs. both sham and Model + BMSCs + PRP.

Based on the above data, p50 could directly upregulate IL-10 expression and secretion induced by IGF-1, whereas IL-10 expression and secretion could be repressed by either inhibition or knockdown of p50. To exclude cell culture-related artifacts, we then attempted to verify this conclusion *in vivo*. For this purpose, p50 stable silencing BMSCs were established, which nearly completely abolished the stimulating effect of PRP in terms of endometrial thickness (Figure 7A). Similarly, p50 silencing obviously suppressed angiogenesis induced by BMSC transplant and PRP therapy (Figure 7B). The α-SMA immunostaining also indicated reduced smooth muscle regeneration compared with control group (Figure 7C). In addition, mRNA level of IGF1 in regenerated endometrium samples was obviously increased by PRP-treated BMSC transplant (Figure 7D, Model + BMSCs + PRP vs. Model), and was unaffected by p50 silencing (Figure 7D, Model + BMSCs/si-p50 + PRP vs. Model + BMSCs + PRP). On the other hand, IL-10 mRNA could also be elevated by PRP-treated BMSC transplant (Figure 7E, Model + BMSCs + PRP vs. Model), but was then significantly repressed by p50 silencing (Figure 7E, Model + BMSCs/si-p50 + PRP vs. Model + BMSCs + PRP). These data demonstrated the indispensable ability of p50 to enhance BMSC transplant following PRP treatment, mediated by direct enhancing of IL-10 production.

## Discussion

**FIGURE 8:**
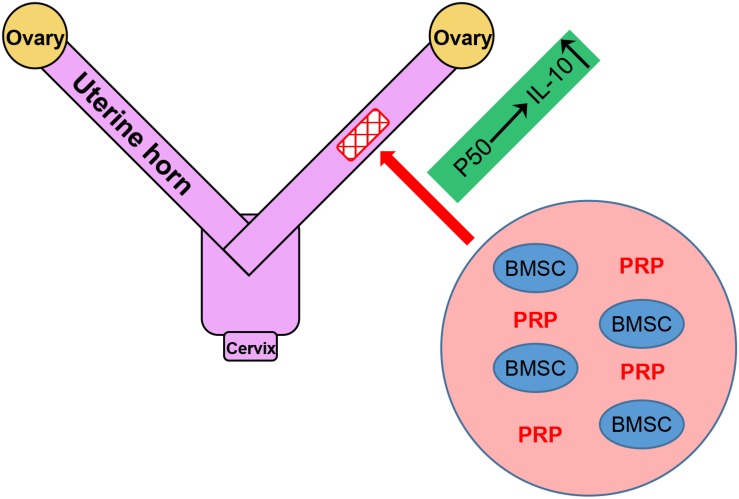
Graphical abstract of the working model.

Endometrial damage is frequent related to infertility and amenorrhea, which heavily weights on the physical health of women worldwide (Di Spiezio Sardo et al., 2016). IGF-1 functions to suppress the release of inflammatory cytokines, the formation of fibrous scars and excessive inflammation in endometrial injury (Ge et al., 2015). In our previous study, we also identified IGF-1 to be the major factor in promoting functional regeneration of injured rat uterus during BMSC transplant (Jin et al., 2016). In the current study, we have brought our discovery further by demonstrating the potent stimulating effect of PRP treatment on BMSC differentiation and IGF-1 expression, which in turn up-regulated IL-10 production and secretion through the NF-κB subunit p50, in rat BMSCs (Figure 3). A working mode is proposed based on results provided in the study, where PRP treatment holds great promise in aiding BMSC transplant for treating uterine horn damage (Figure 8).

The effectiveness of PRP therapy on uterus-related diseases have been documented. For example, Chrysanthopoulou et al. (2017) suggested that PRP could be a minimally invasive method against uterine prolapse. In the rat model of intrauterine adhesion, it has been found that PRP could enhance the therapy of stromal cells derived from menstrual blood to repair endometrial damage (Zhang et al., 2019). To bring their discoveries further, our current study serves as one novel instance, where PRP exhibits previously unknown synergistic effect on BMSCs to enhance endometrial regeneration of injured uterus in rats, suggesting that the therapeutic usefulness of PRP could be wider than previously thought and worth further investigations in even more types of disease models. Moreover, based on results from our experiments, the above-mentioned beneficial effects of PRP treatment are likely mediated by increasing the tri-lineage potential and promoting IL-10 expression and secretion of BMSCs. In order to provide more solid evidence to support the role of PRP-treatment on transplanted BMSCs, paracrine factors secreted by the transplated BMSCs during the process of uterine wound regeneration should be investigated in future study.

To better understand the beneficial effect of PRP on BMSC transplant, we attempted to reveal the machinery by which PRP up-regulates IL-10 production. Next, a putative recognition site of p50 was identified on IL-10 promoter, and was experimentally validated offer direct binding of p50. As p50 is a transcriptional factor, its direct binding to a *cis-*element would facilitate the transcription of a downstream gene, the anti-inflammatory cytokine IL-10 in our case. A number of previous studies have established that IL-10 is under IGF-10 stimulation in various cell types (Warzecha et al., 2003; Kooijman and Coppens, 2004; Strle et al., 2008; Lu et al., 2014). Our observation that, although p50 directly up-regulated IL-10, its inhibition or knockdown did not affect IGF-1, has therefore established a signaling cascade in the order of IGF-1/p50/IL-10. To further support this notion, the effect of IGF-1 silencing on IL-10 expression and secretion would be investigated in future study.

In our current experimental setting, a special attention was paid to the role of NF-κB p50 subunit, in activating the transcription of IL-10. This observation has been consolidated by accumulating evidences, demonstrating the regulating effect of NF-κB subunit p50 on the expression of IL-10 in different types of cells. For example, Dibra et al. demonstrated the synergistical effect of T cells and macrophages on enhancing IL-10 through NF-κB (Dibra and Li, 2013). In addition, data in our study again clearly indicated that p50 could obviously activate IL-10, which was in agreement with Cao et al’s. (2006) study in which p50 homodimers showed specific binding to the −55/−46 locus in the IL-10 promoter to induce its expression. The anti-inflammatory cytokine IL-10 has been reported to be involved in uterine horn damage. In rats receiving ovariectomy (OV) to induce uterine horn damage, reduced IL-10 level was observed in the uterus horn tissue of OV rats, which could be rescued by estrogen treatment, implicating IL-10 in the damage and, in a more important sense, the repair of uterine horn damage (Cevik et al., 2015). More recently, Xue et al. (2019) reported that leukemia inhibitory factor could promote rat uterine horn regeneration by up-regulating IL-10 expression in the injured part of the uterine horns. Results in our study have proposed, for the first time, the upstream regulatory mechanism of IL-10 in aiding the repair of uterine horn damage: in response to IGF-1 overexpression in the inflammatory setting, the NF-κB pathway is activated via the p50 subunit, and the transcriptional factor p50 subunit is translocated into the nucleus, where it specifically binds to IL-10 promoter to activate its transcription (Figure 8). These novel findings also highlight p50/IL-10 as potential therapeutic targets for future treatments against uterine horn damage.

To sum up, our current results give a clear demonstration of the obvious beneficial effect of PRP treatment on BMSC transplant. In terms of mechanism, PRP treatment enhances BMSC differentiation, induces IGF-1 expression and activates the NF-κB pathway subunit p50, which in turn elevates the expression and production of IL-10 by directly binding to its promoter. The strategy proposed in the current study provides a foundation for later investigations aiming to optimize the clinical efficacy of BMSC transplant for the repair of uterine horn damage.

## Data Availability Statement

Data could be obtained upon reasonal request to the corresponding author.

## Ethics Statement

This study was carried out in accordance with the recommendations of the Ethics Commitment of Shanghai First Maternity and Infant Hospital, Tongji University School of Medicine. This animal study strictly followed the instruction of the Animal Care and Use Committee in Shanghai First Maternity and Infant Hospital, and approved by the Ethics Commitment of Shanghai First Maternity and Infant Hospital, Tongji University School of Medicine (Approval No. SFMIH5314K-04).

## Author Contributions

YZ, HS, YW, XZ, JP, ZM, JD, and XH performed the experiments and analyzed and interpreted the data. XH were the major contributors in writing the manuscript. All authors read and approved the final manuscript.

## Conflict of Interest

The authors declare that the research was conducted in the absence of any commercial or financial relationships that could be construed as a potential conflict of interest.
