# Supplementary material for: Platelet-Rich Plasma Therapy Enhances the Beneficial Effect of Bone Marrow Stem Cell Transplant on Endometrial Regeneration
Source: Front Cell Dev Biol. 2020 Feb 21;8:52. doi: 10.3389/fcell.2020.00052 (PMC7047166; doi:10.3389/fcell.2020.00052)

Supplementary materials

Supplementary Figure 1. Full scans of the entire original gels used in Figure 3C.

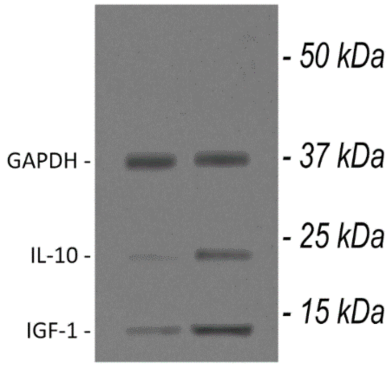

Supplementary Figure 2. Full scans of the entire original gels used in Figure 4A.

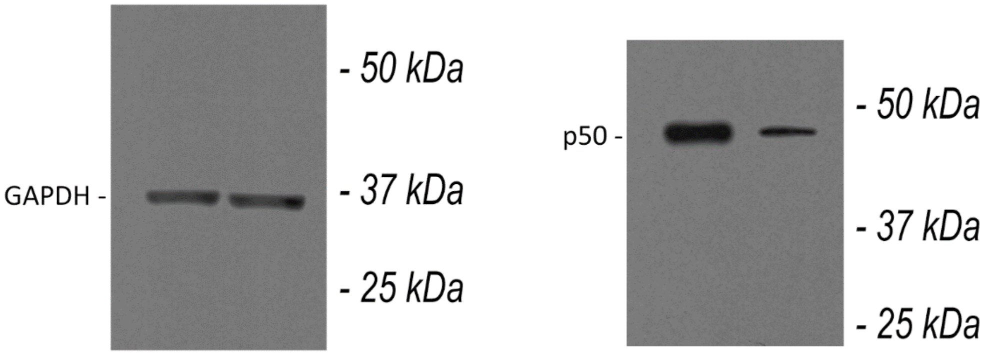

Supplementary Figure 3. Full scans of the entire original gels used in Figure 5C.

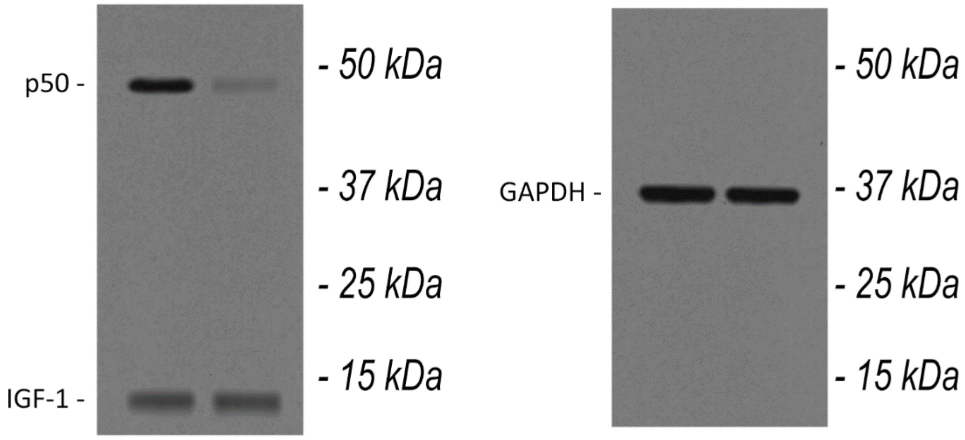

Supplement: Supplementary file 1 [file Data_Sheet_1.pdf]
